# Knowledge, attitudes, and practices of nurses regarding bowel preparation for colonoscopy: a structural equation modeling analysis

**DOI:** 10.3389/fpubh.2026.1921528

**Published:** 2026-09-14

**Authors:** Qian Han, Hongping Zhou, Lin Xu, Huiming Tu

**Affiliations:** Endoscopy Center, Affiliated Hospital of Jiangnan University, Wuxi, Jiangsu, China

**Keywords:** attitudes, bowel preparation, colonoscopy, knowledge, nurses, practice

## Abstract

**Objective:**

This study aimed to investigate nurses' knowledge, attitude, and practice regarding bowel preparation for colonoscopy.

**Methods:**

This multicenter cross-sectional study was conducted among registered nurses from eight hospitals in Jiangsu Province, China, between December 2025 and March 2026. Data were collected using a self-administered online questionnaire covering demographic characteristics and KAP scores. Multivariable linear regression was used to identify factors associated with KAP scores, and structural equation modeling (SEM) was used to explore the relationships among knowledge, attitudes, and practices.

**Results:**

A total of 382 valid questionnaires were included. The median knowledge, attitude, and practice scores were 10.00 (9.00, 14.00), 37.00 (36.00, 43.00), and 28.00 (23.00, 32.00), respectively. Lack of bowel preparation-related training was independently associated with lower knowledge (β = −4.097, 95% CI: −5.006 to −3.188), attitude (β = −1.052, 95% CI: −2.000 to −0.104), and practice scores (β = −2.297, 95% CI: −3.335 to −1.260). SEM showed significant direct associations of knowledge with attitude (β = 0.491, 95% CI: 0.407–0.584, *P* = 0.001) and practice (β = 0.707, 95% CI: 0.626–0.780, *P* = 0.002), as well as a significant indirect association between knowledge and practice through attitude (β = 0.068, 95% CI: 0.024–0.118, *P* = 0.002).

**Conclusion:**

Nurses demonstrated insufficient knowledge, positive attitudes, and moderate practice regarding bowel preparation for colonoscopy. These findings identify potential targets for future nurse-oriented educational and organizational interventions, particularly knowledge gaps and bowel preparation-related practices.

## Introduction

Colorectal cancer (CRC) represents a major global public health challenge, with an estimated 1.9 million new cases and approximately 900,000 deaths annually, accounting for 9.3% of all cancer-related mortality worldwide (1). In China, the disease burden of CRC has been particularly concerning, with the age-standardized incidence rate increasing at an average annual percentage change of 1.66% from 1990 to 2021, and the cancer now ranking as the second most common malignancy and the fourth leading cause of cancer death nationwide (2). Early detection through colonoscopy screening has been unequivocally established as a cornerstone strategy for reducing both the incidence and mortality of CRC. The removal of precancerous adenomas during colonoscopy can effectively interrupt the natural progression from adenoma to carcinoma, while early-stage cancer detection enables curative intervention. Current clinical guidelines from major gastroenterological societies worldwide recommend colonoscopy as the preferred screening modality for average-risk individuals beginning at age 45 to 50 years (3, 4).

Importantly, the effectiveness of colonoscopy depends heavily on the quality of bowel preparation. Inadequate bowel cleansing can reduce mucosal visualization, compromise adenoma detection, prolong procedure time, increase the need for repeat examinations, and ultimately weaken the preventive value of colonoscopy (5, 6). Recent quality indicators for colonoscopy continue to identify adequate bowel preparation as a core prerequisite for high-quality examination (6). Similarly, updated bowel preparation guidelines recommend enhanced patient instructions, split-dose regimens, appropriate dietary modification, and systematic assessment of preparation quality (4, 7). These recommendations indicate that bowel preparation is not merely a patient-dependent behavior but a coordinated clinical process requiring standardized education, monitoring, and multidisciplinary support.

Nurses are closely involved in patient education, medication instruction, dietary guidance, assessment of adherence, and communication with physicians when bowel preparation is inadequate. Previous interventional studies and reviews have shown that reinforced education, particularly nurse-led or technology-assisted education, can improve the adequacy of bowel preparation and colonoscopy-related quality outcomes (8–10). However, the translation of guideline recommendations into routine nursing practice depends on nurses' knowledge of bowel preparation standards, their attitudes toward the importance of bowel preparation management, and their actual clinical behaviors. The knowledge–attitude–practice (KAP) theoretical model posits that knowledge shapes attitudes, which in turn influence practices (11, 12). KAP-based studies are widely used to identify discrepancies between evidence-based recommendations and routine clinical practice, thereby informing targeted educational and organizational interventions (13). To the best of our knowledge, no prior study has specifically examined nurses' knowledge, attitudes, and practices regarding bowel preparation for colonoscopy, despite the recognized importance of nurses' involvement in this process, particularly in the Chinese clinical context. Existing KAP studies related to colonoscopy have primarily considered colonoscopy as part of colorectal cancer screening (14–16), rather than focusing on bowel preparation or nurses' role in patient education and preparation management.

Accordingly, this study aimed to investigate the knowledge, attitudes, and practices of nurses regarding bowel preparation for colonoscopy and to examine the relationships among these dimensions.

## Methods

### Study design and participants

This multicenter cross-sectional study was conducted between December 2025 and March 2026 across eight hospitals in Jiangsu Province, China. The study population comprised registered nurses working in clinical settings. Ethical approval was obtained from the Medical Ethics Committee of Jiangnan University Affiliated Hospital, and all participants provided informed consent before data collection. Eligible participants were registered nurses who: (1) held a valid nursing practice license; (2) had at least 6 months of clinical nursing experience; (3) had provided care for patients undergoing colonoscopy; and (4) were informed of the study objectives and voluntarily agreed to participate. Nursing students, interns, trainee nurses, and unregistered temporary staff were excluded to ensure that the study population consisted only of professionally qualified clinical nurses. The participating hospitals were located in Jiangsu Province, where endoscopy services are generally covered by basic medical insurance. Endoscopy-center nurses are routinely responsible for pre-procedural education and bowel-preparation guidance, endoscope cleaning and disinfection, procedural assistance, and post-procedural safety management. Before independent practice, nurses generally complete at least 3 months of specialized training in endoscope reprocessing and endoscopic procedural assistance, while basic endoscopy-related knowledge is included in pre-licensure nursing education. The participating hospitals generally followed consistent clinical guidelines, procedural standards, and assessment tools for endoscopy-related nursing practice.

### Questionnaire

The questionnaire was developed on the basis of domestic and international relevant clinical guidelines, including the *Consensus on bowel preparation for colonoscopy (2023, Guangzhou)* (17), the *Guidelines for Bowel Preparation Related to Digestive Endoscopy in China (2019, Shanghai)* (18), and the *Bowel preparation for colonoscopy: European Society of Gastrointestinal Endoscopy (ESGE) Guideline* (4). Following completion of the initial draft, expert consultation was undertaken with three nursing experts, each with more than 20 years of professional experience. Based on their feedback, the questionnaire was further refined by deleting semantically redundant items, supplementing necessary content, and standardizing the professional wording of the item descriptions. A pilot survey involving 35 participants was subsequently conducted to confirm the reliability of the instrument. The Cronbach's α coefficients were 0.932 for the knowledge dimension, 0.925 for the attitude dimension, 0.877 for the practice dimension, and 0.942 for the overall KAP scale, indicating good internal consistency. During the pilot survey, participants were also asked to provide feedback on the clarity and comprehensibility of the questionnaire items. No difficulties in understanding the wording or content of the items were reported, thereby supporting the face validity of the questionnaire.

The final questionnaire was administered in Chinese and comprised four sections: demographic characteristics, knowledge, attitude, and practice. An English-translated version of the questionnaire is provided in the Additional File of Questionnaire. Demographic information included age, sex, educational level, work experience, professional title, department, hospital level, and training experience related to bowel preparation during the past year. The knowledge dimension consisted of 10 items, scored as 2 points for “very familiar,” 1 point for “heard of it,” and 0 points for “do not know,” yielding a total score ranging from 0 to 20. The attitude dimension included 9 items rated on a five-point Likert scale from 5 (“strongly agree”) to 1 (“strongly disagree”), with a total score ranging from 9 to 45. The practice dimension comprised 8 items scored from 1 (“never”) to 5 (“always”), yielding a total score ranging from 8 to 40. Consistent with the Bloom's cut-off criteria commonly applied in KAP studies (19, 20), scores for each dimension were categorized according to the percentage of the maximum attainable score: < 60% was defined as poor, 60%−79% as moderate, and ≥80% as good.

### Questionnaire distribution and quality control

A convenience sampling approach was used, and the questionnaire was administered online. Participants were contacted via WeChat, and the online questionnaire link was distributed through WeChat groups. Before accessing the questionnaire, participants were presented with a brief introduction describing the study purpose, study content, anonymity, privacy protection principle, and voluntary nature of participation. Only those who finished reading the information and provided informed consent proceeded to the questionnaire, while those who declined consent were directed to exit the survey. Two trained research assistants facilitated the survey process and were available online to answer questions regarding the study purpose or procedures. To minimize missing data, all questionnaire items were set as mandatory before submission. To enhance data quality, predefined screening criteria were applied to identify and exclude invalid questionnaires or responses with potential information or selection bias. First, an attention-check question (“10 + 10 = 30”) was included to identify inattentive or random responses. Second, questionnaires completed in less than 60 s were excluded, as this duration was considered insufficient for careful reading and thoughtful responses. Third, questionnaires containing obvious numerical anomalies, such as an age of 187 years, were removed to minimize systematic error. Fourth, questionnaires showing repetitive response patterns, such as selecting the same option for all items, were excluded because these patterns suggested insufficient engagement.

### Sample size

The sample size was calculated using the formula for cross-sectional studies (21): *n* = *Z*^2^*p*(1–*p*)/*e*^2^. In this formula, n represents the required sample size, Z represents the standard normal deviation corresponding to a 95% confidence level (*Z* = 1.96), p represents the expected proportion, and e represents the allowable margin of error (set at 5%). Considering no previous studies have specifically evaluated bowel preparation–related KAP level among nurses, a conservative estimate of *p* = 0.50 was adopted to maximize the required sample size. Accordingly, the minimum estimated sample size was 384 participants. The final analytic sample included 382 valid questionnaires, corresponding to an achieved precision of approximately 5.01%.

### Statistical analysis

Statistical analyses were performed using R software version 4.4.3 and AMOS version 24.0 (IBM, Armonk, NY, USA). The normality of continuous variables was assessed using the Kolmogorov–Smirnov test and *Q*–*Q* plots. As the KAP scores were deviated from normality and were therefore summarized as medians with interquartile ranges [*M* (Q1, Q3)]. Between-group comparisons for skewed continuous variables were conducted using the Wilcoxon–Mann–Whitney *U*-test for two groups and the Kruskal–Wallis test for multiple groups. Categorical variables were presented as *n* (%). Spearman's correlation analysis was used to evaluate correlations among KAP scores. To identify factors independently associated with KAP scores, univariable and multivariable linear regression analyses were performed. Variables with *P* < 0.05 in univariable analyses, together with variables considered clinically relevant based on prior literature and subject-matter knowledge, were included in the multivariable models. The final multivariable model for knowledge included educational level, department, work experience, hospital level, research responsibilities, and bowel preparation-related training. The model for attitude included educational level, department, bowel preparation-related training, and knowledge score; and the model for practice included knowledge score, attitude score, age, department, work experience, hospital level, research responsibilities, and bowel preparation-related training. Based on the KAP theoretical framework, structural equation modeling (SEM) was conducted to examine the hypothesized direct and indirect associations among knowledge, attitude, and practice, including the indirect association between knowledge and practice through attitude. SEM was performed using maximum likelihood estimation in AMOS. Multivariate normality was assessed before model estimation. Given evidence of multivariate non-normality (multivariate kurtosis = 342.426; critical ratio = 84.561), bias-corrected bootstrap procedures with 1,000 resamples were used to obtain 95% confidence intervals for the structural associations. Model fit was evaluated using the root mean square error of approximation (RMSEA), chi-square/degrees of freedom ratio (CMIN/DF), Tucker–Lewis index (TLI), and comparative fit index (CFI). Model fit was considered acceptable when RMSEA < 0.08, CMIN/DF < 5.00, and CFI and TLI ≥ 0.90 (22, 23). Common method bias was additionally assessed using Harman's single-factor analysis. All 27 KAP items were entered simultaneously into an unrotated factor solution, and the proportion of total variance explained by the first factor was examined. A first-factor variance below 50% was considered to indicate that no single common factor dominated the covariance among the questionnaire items. A two-sided *P* < 0.05 was considered statistically significant.

## Results

### Basic characteristics of the included nurses

A total of 435 questionnaires were initially collected. After data screening, 53 responses were excluded, including 7 without informed consent, 1 with a response time of less than 60 s, 42 with incorrect answers to attention-check questions, and 3 with implausible age values. Finally, 382 valid questionnaires were included in the analysis, yielding a valid questionnaire rate of 87.8%. Among the included nurses, 68 (17.8%) were male and 314 (82.2%) were female. The majority of participants were aged ≤ 34 years (39.3%), held a bachelor's degree (82.7%), worked in other departments (42.4%), had 11–20 years of work experience (46.3%), and held an intermediate professional title (58.6%). Most were employed in public hospitals (94.0%) and tertiary hospitals (87.4%), and had not received bowel preparation-related training during the past year (58.4%) (Table 1).

**Table 1:** Basic characteristics of included nurses.

Variable	*N* (%)	Knowledge, *M* (*Q*1, *Q*3)	*P*	Attitude, *M* (*Q*1, *Q*3)	*P*	Practice, M (Q1, Q3)	*P*
**Total score**	382 (100.0%)	10.00 (9.00, 14.00)		37.00 (36.00, 43.00)		28.00 (23.00, 32.00)	
**Sex**			0.173		0.040		0.366
Male	68 (17.8%)	11.00 (8.75, 16.00)		39.00 (36.00, 43.25)		30.00 (24.00, 34.00)	
Female	314 (82.2%)	10.00 (9.00, 14.00)		36.00 (36.00, 43.00)		28.00 (23.00, 32.00)	
**Age (years)**			0.507		0.690		0.026
≤ 34 years	150 (39.3%)	10.00 (8.25, 14.00)		37.00 (36.00, 42.75)		29.00 (24.00, 32.00)	
35–39 years	135 (35.3%)	10.00 (8.50, 13.50)		37.00 (35.00, 43.00)		26.00 (22.00, 31.50)	
≥40 years	97 (25.4%)	10.00 (9.00, 15.00)		37.00 (36.00, 43.00)		30.00 (24.00, 33.00)	
**Educational level**			0.215		0.082		0.340
Junior college	28 (7.3%)	10.00 (8.00, 17.25)		36.50 (36.00, 43.25)		28.50 (24.00, 37.25)	
Bachelor's degree	316 (82.7%)	10.00 (9.00, 14.00)		37.00 (35.75, 42.00)		28.00 (23.00, 32.00)	
Master's degree or above	38 (9.9%)	11.00 (9.00, 19.00)		39.50 (36.00, 43.00)		30.00 (25.00, 35.00)	
**Department**			< 0.001		0.066		0.002
Endoscopy center	41 (10.7%)	12.00 (10.00, 19.00)		40.00 (36.00, 43.00)		31.00 (27.00, 35.00)	
Department of Gastroenterology	132 (34.6%)	10.00 (9.00, 16.00)		38.00 (36.00, 44.00)		29.50 (24.00, 32.25)	
General Surgery Department	47 (12.3%)	10.00 (5.00, 11.50)		36.00 (35.00, 40.00)		25.00 (19.00, 31.00)	
Other departments	162 (42.4%)	10.00 (8.00, 13.00)		36.00 (35.00, 42.00)		27.50 (23.00, 32.00)	
**Work experience (years)**			0.033		0.205		0.044
≤ 5	68 (17.8%)	10.00 (9.75, 14.25)		37.00 (35.75, 43.00)		30.00 (25.00, 32.25)	
5–10	86 (22.5%)	10.00 (8.25, 16.00)		37.50 (36.00, 42.00)		26.00 (23.00, 32.00)	
11–20	177 (46.3%)	10.00 (8.00, 13.00)		36.00 (35.00, 43.00)		28.00 (22.00, 32.00)	
≥21	51 (13.4%)	11.00 (10.00, 16.50)		37.00 (36.00, 43.00)		30.00 (24.00, 34.00)	
**Professional title**			0.584		0.347		0.577
Junior title or below	93 (24.3%)	10.00 (9.00, 15.00)		36.00 (36.00, 42.00)		29.00 (24.00, 32.00)	
Intermediate title	224 (58.6%)	10.00 (8.00, 14.00)		37.00 (35.00, 42.00)		28.00 (23.00, 32.00)	
Senior title, including associate senior title	65 (17.0%)	10.00 (10.00, 14.00)		37.00 (36.00, 44.00)		28.00 (24.00, 31.00)	
**Hospital type**			0.354		0.149		0.769
Private hospital	23 (6.0%)	10.00 (9.00, 17.50)		38.00 (36.00, 44.50)		28.00 (23.00, 34.50)	
Public hospital	359 (94.0%)	10.00 (9.00, 14.00)		37.00 (36.00, 43.00)		28.00 (23.00, 32.00)	
**Hospital level**			0.018		0.063		0.001
Non-tertiary hospital	48 (12.6%)	10.50 (10.00, 18.00)		38.00 (36.00, 43.00)		32.00 (25.00, 38.00)	
Tertiary hospital	334 (87.4%)	10.00 (8.25, 14.00)		37.00 (35.25, 43.00)		28.00 (23.00, 32.00)	
**Teaching responsibilities**			0.227		0.184		0.878
No	178 (46.6%)	10.00 (9.00, 13.00)		36.00 (36.00, 42.00)		28.00 (24.00, 32.00)	
Yes	204 (53.4%)	10.00 (9.00, 14.25)		37.00 (36.00, 43.00)		29.00 (23.00, 32.00)	
**Research responsibilities**			0.014		0.294		0.046
No	270 (70.7%)	10.00 (8.25, 14.00)		37.00 (36.00, 42.00)		28.00 (23.00, 32.00)	
Yes	112 (29.3%)	11.00 (9.00, 16.00)		38.00 (35.75, 43.00)		30.00 (24.00, 34.00)	
**Monthly colonoscopy-related nursing frequency in the past year (cases)**			0.523		0.649		0.266
< 10	60 (15.7%)	10.00 (8.00, 14.00)		37.00 (36.00, 43.00)		28.00 (23.00, 32.00)	
10–20	116 (30.4%)	10.00 (9.00, 13.00)		37.00 (35.00, 41.25)		26.00 (23.00, 31.25)	
21–30	136 (35.6%)	10.00 (9.00, 16.00)		37.00 (36.00, 42.25)		30.00 (24.00, 32.00)	
>30	70 (18.3%)	10.00 (9.00, 16.25)		37.50 (36.00, 44.75)		29.00 (22.00, 32.00)	
**Training related to bowel preparation during the past year**			< 0.001		< 0.001		< 0.001
No	223 (58.4%)	10.00 (7.00, 11.00)		36.00 (35.00, 40.00)		25.00 (20.00, 30.50)	
Yes	159 (41.6%)	13.00 (10.00, 19.00)		40.00 (36.00, 44.00)		32.00 (27.00, 36.50)	

Data are presented as *n* (%) for categorical variables and median (Q1, Q3) for knowledge, attitude, and practice scores. Between-group comparisons were performed using the Wilcoxon–Mann–Whitney U-test for two-group variables and the Kruskal–Wallis test for variables with more than two groups. KAP, knowledge, attitude, and practice; Q1, first quartile; Q3, third quartile.

The median knowledge, attitude, and practice scores were 10.00 (Q1, Q3: 9.00, 14.00), 37.00 (36.00, 43.00), and 28.00 (23.00, 32.00), respectively, indicating insufficient knowledge, generally positive attitudes, and moderate practice according to Bloom's cut-off criteria. Nurses who had received bowel preparation-related training during the past year had higher knowledge, attitude, and practice scores than those who had not received such training (all *P* < 0.001). Also, significant differences in knowledge scores were observed across department, work experience, hospital level, research responsibilities, and training related to bowel preparation during the past year (all *P* < 0.05). Attitude scores differed significantly by sex and training status, whereas practice scores differed significantly by age, department, work experience, hospital level, research responsibilities, and training status (all *P* < 0.05; Table 1).

### Item-level response distribution of KAP dimensions

For the knowledge dimension, the most frequently unrecognized item was K3, which concerned the criterion for adequate bowel preparation based on the Boston Bowel Preparation Scale, with 147 nurses (38.5%) selecting “Do not know.” Other knowledge gaps were observed for items related to commonly used bowel-cleansing agents (K5: 62, 16.2%), dietary restrictions before colonoscopy (K8: 61, 16.0%), and risk factors for inadequate bowel preparation (K9: 57, 14.9%) (Table 2).

**Table 2:** Distribution of knowledge dimension responses.

Knowledge items, *n* (%)	Very familiar	Heard of it	Do not know
1. Adequate bowel preparation is a prerequisite for high-quality colonoscopy.	94 (24.6%)	245 (64.1%)	43 (11.3%)
2. Inadequate bowel preparation may increase the risk of missed lesions during colonoscopy.	80 (20.9%)	246 (64.4%)	56 (14.7%)
3. A Boston Bowel Preparation Scale (BBPS) score of ≥6 is generally considered to indicate adequate bowel preparation.	53 (13.9%)	182 (47.6%)	147 (38.5%)
4. Split-dose laxative administration is more effective than single-dose administration.	166 (43.5%)	194 (50.8%)	22 (5.8%)
5. Polyethylene glycol (PEG) electrolyte powder is currently the most commonly used bowel cleansing agent in China.	117 (30.6%)	203 (53.1%)	62 (16.2%)
6. Patient adherence affects the effectiveness of bowel preparation.	99 (25.9%)	241 (63.1%)	42 (11.0%)
7. Oral sulfate solution (OSS) is a novel bowel preparation agent that can induce rapid defecation.	94 (24.6%)	237 (62.0%)	51 (13.4%)
8. A low-residue/low-fiber diet helps improve bowel preparation, and high-residue/high-fiber foods should be avoided within 24 h before colonoscopy.	96 (25.1%)	225 (58.9%)	61 (16.0%)
9. Older age, obesity, chronic constipation, or chronic diseases increase the risk of inadequate bowel preparation.	107 (28.0%)	218 (57.1%)	57 (14.9%)
10. Health education can improve the success rate of bowel preparation.	124 (32.5%)	216 (56.5%)	42 (11.0%)

Data are presented as *n* (%).

Regarding attitudes, the highest combined proportion of “Disagree” or “Strongly disagree” was found for A2, which reflected nurses' perceived role in bowel preparation, with 37 participants (9.7%) expressing disagreement. This was followed by items concerning the perceived impact of bowel preparation quality on colonoscopy results (A1: 10, 2.6%), the potential role of bowel preparation education in reducing patient anxiety (A8: 7, 1.8%), routine recommendation of split-dose medication (A6: 5, 1.3%), and patient education as a key strategy for improving bowel preparation quality (A3: 3, 0.8%) (Table 3).

**Table 3:** Distribution of attitude dimension responses.

Attitude items, *n* (%)	Strongly agree	Agree	Neutral	Disagree	Strongly disagree
1. I believe that the quality of bowel preparation directly affects colonoscopy results.	132 (34.6%)	205 (53.7%)	35 (9.2%)	9 (2.4%)	1 (0.3%)
2. I believe that nurses play an important role in bowel preparation.	87 (22.8%)	195 (51.0%)	63 (16.5%)	36 (9.4%)	1 (0.3%)
3. I believe that patient education is key to improving the quality of bowel preparation.	155 (40.6%)	207 (54.2%)	17 (4.5%)	3 (0.8%)	0 (0.0%)
4. I believe that all patients should receive systematic education, including combined oral, written, and electronic education.	158 (41.4%)	211 (55.2%)	11 (2.9%)	1 (0.3%)	1 (0.3%)
5. I believe that the bowel preparation process should be standardized.	149 (39.0%)	213 (55.8%)	18 (4.7%)	1 (0.3%)	1 (0.3%)
6. I believe that split-dose medication should be routinely recommended.	152 (39.8%)	203 (53.1%)	22 (5.8%)	5 (1.3%)	0 (0.0%)
7. I believe that management of high-risk patients should be strengthened.	159 (41.6%)	195 (51.0%)	25 (6.5%)	1 (0.3%)	2 (0.5%)
8. I believe that effective bowel preparation education can reduce patients' anxiety.	131 (34.3%)	212 (55.5%)	32 (8.4%)	4 (1.0%)	3 (0.8%)
9. I am willing to participate in training and learning related to bowel preparation.	146 (38.2%)	205 (53.7%)	29 (7.6%)	2 (0.5%)	0 (0.0%)

Data are presented as *n* (%).

Practice responses indicated that the least active behaviors were mainly related to individualized guidance, documentation, reporting, and patient consultation. Specifically, 211 participants (55.2%) reported that they rarely or never provided individualized guidance for high-risk patients (P2), 107 (28.0%) rarely or never recorded patients' bowel cleanliness status after bowel preparation (P7), and 100 (26.2%) rarely or never proactively reported inadequate bowel preparation to physicians and assisted in subsequent management adjustment (P8) (Table 4).

**Table 4:** Distribution of practice dimension responses.

Practice items, *n* (%)	Always	Often	Sometimes	Rarely	Never
1. I explain the importance of bowel preparation to patients.	54 (14.1%)	156 (40.8%)	107 (28.0%)	58 (15.2%)	7 (1.8%)
2. I provide individualized guidance for high-risk patients.	38 (9.9%)	58 (15.2%)	75 (19.6%)	114 (29.8%)	97 (25.4%)
3. I explain in detail how to use laxatives.	167 (43.7%)	118 (30.9%)	52 (13.6%)	37 (9.7%)	8 (2.1%)
4. I provide dietary guidance to patients.	115 (30.1%)	160 (41.9%)	73 (19.1%)	32 (8.4%)	2 (0.5%)
5. I remind patients to complete bowel preparation on time.	101 (26.4%)	141 (36.9%)	97 (25.4%)	40 (10.5%)	3 (0.8%)
6. I answer patients' questions related to bowel preparation.	84 (22.0%)	130 (34.0%)	95 (24.9%)	61 (16.0%)	12 (3.1%)
7. I record patients' bowel cleanliness status after bowel preparation.	65 (17.0%)	118 (30.9%)	92 (24.1%)	75 (19.6%)	32 (8.4%)
8. I proactively report inadequate bowel preparation to physicians and assist in adjusting subsequent management plans.	70 (18.3%)	123 (32.2%)	89 (23.3%)	84 (22.0%)	16 (4.2%)

Data are presented as *n* (%).

### Factors associated with KAP scores

The results of multivariable linear regression analysis revealed that knowledge scores were significantly lower among nurses without bowel-preparation-related training (β = −4.097, 95% CI: −5.006 to −3.188, *P* < 0.001), those in the General Surgery Department (β = −2.812, 95% CI: −4.681 to −0.943, *P* = 0.003) or other departments (β = −2.071, 95% CI: −3.611 to −0.532, *P* = 0.008), those with 11–20 years of work experience (β = −1.480, 95% CI: −2.772 to −0.188, *P* = 0.025), those in tertiary hospitals (β = −1.795, 95% CI: −3.125 to −0.465, *P* = 0.008), and those without research responsibilities (β = −1.102, 95% CI: −2.129 to −0.075, *P* = 0.035). Attitude scores were significantly lower among nurses without bowel-preparation-related training (β = −1.052, 95% CI: −2.000 to −0.104, *P* = 0.030), whereas higher knowledge scores were independently associated with more positive attitudes (β = 0.381, 95% CI: 0.287 to 0.476, *P* < 0.001). Also, practice scores were significantly lower among nurses without bowel-preparation-related training (β = −2.297, 95% CI: −3.335 to −1.260, *P* < 0.001), those with 5–10 years of work experience (β = −1.787, 95% CI: −3.275 to −0.299, *P* = 0.019), and those in tertiary hospitals (β = −1.411, 95% CI: −2.803 to −0.020, *P* = 0.047), while higher knowledge (β = 0.781, 95% CI: 0.668 to 0.894, *P* < 0.001) and attitude scores (β = 0.223, 95% CI: 0.114 to 0.331, *P* < 0.001) were independently associated with better practice (Supplementary Tables S1–S3).

### Correlations among knowledge, attitude, and practice scores

Spearman correlation analysis showed that knowledge score was positively correlated with attitude score (*r* = 0.434, *P* < 0.001) and practice score (*r* = 0.707, *P* < 0.001). Attitude score was also positively correlated with practice score (*r* = 0.471, *P* < 0.001; Supplementary Table S4).

### SEM analysis

Harman's single-factor analysis identified four factors with eigenvalues greater than 1, with the first factor accounting for 44.58% of the total variance. As this proportion was below 50%, no single factor appeared to dominate the covariance among the KAP items. The SEM analysis showed acceptable model fit. The CMIN/DF was 2.941, RMSEA was 0.071, TLI was 0.911, and CFI was 0.920, indicating that the hypothesized KAP model adequately fitted the observed data (Supplementary Table S5). In the structural model, knowledge showed significant positive association with attitude (β = 0.491, 95% CI: 0.407–0.584, *P* = 0.001) and practice (β = 0.707, 95% CI: 0.626–0.780, *P* = 0.002). In addition, attitude had a significant positive association with practice (β = 0.139, 95% CI: 0.045–0.230, *P* = 0.003). Furthermore, a significant indirect association between knowledge and practice through attitude was observed (β = 0.068, 95% CI: 0.024–0.118, *P* = 0.002; Table 5 and Figure 1). The measurement model further showed that all observed indicators loaded significantly onto their corresponding latent constructs (Supplementary Table S6).

**Table 5:** Bootstrap analysis of standardized direct, indirect, and total associations in the structural equation model.

Model paths	Total association		Direct association		Indirect association	
	β (95%CI)	*P*	β (95%CI)	*P*	β (95%CI)	*P*
Knowledge → Attitude	0.491 (0.407–0.584)	0.001	0.491 (0.407–0.584)	0.001	—	—
Knowledge → Practice	0.775 (0.726–0.825)	0.001	0.707 (0.626–0.780)	0.002	0.068 (0.024–0.118)	0.002
Attitude → Practice	0.139 (0.045–0.230)	0.003	0.139 (0.045–0.230)	0.003	—	—

β represents the standardized path coefficient. Confidence intervals were estimated using the bias-corrected bootstrap method with 1,000 resamples. The indirect association between knowledge and practice through attitude was estimated within the hypothesized structural model. CI, confidence interval. “—” indicates that the corresponding indirect association was not applicable.

**Figure 1:**
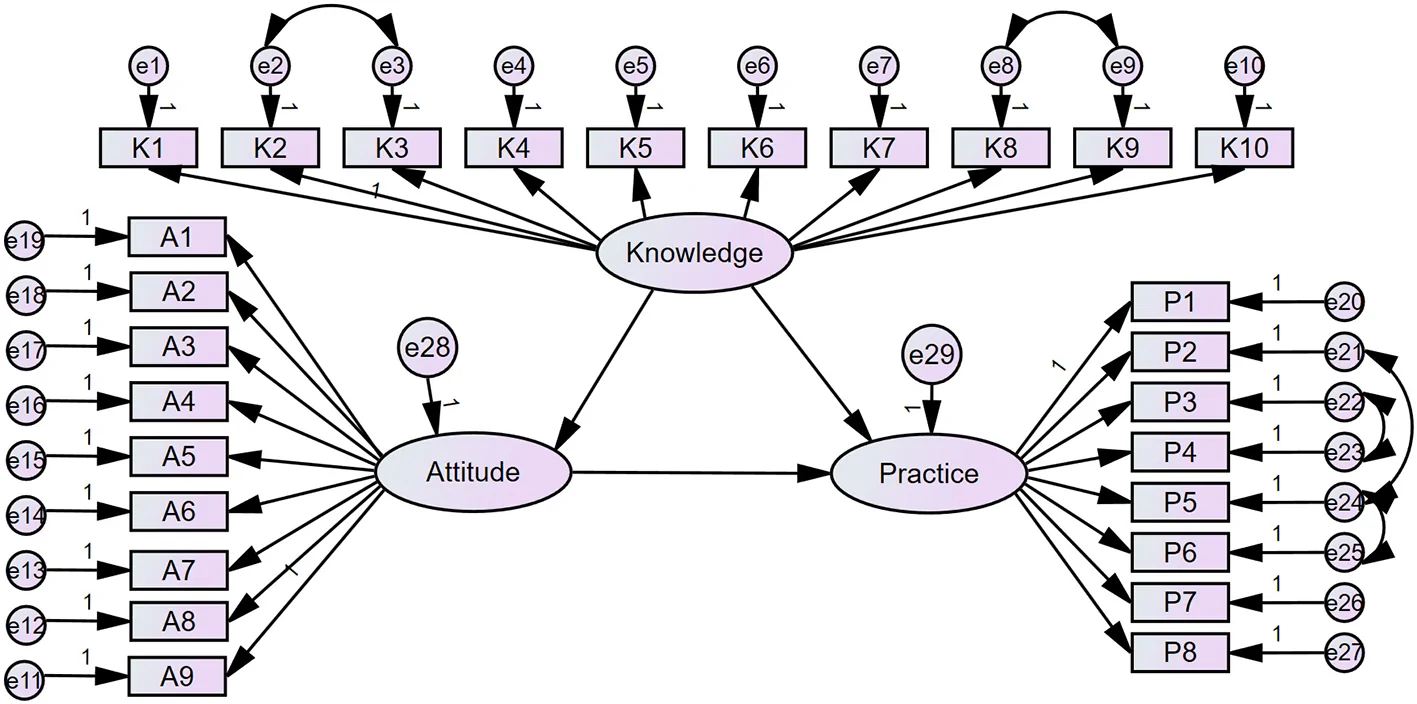
Structural Equation Model (SEM) depicting the relationships among the latent variables: knowledge, attitude, and practice. Ellipses represent latent variables (unobserved constructs), including overall knowledge, attitude, and practice. Rectangles represent observed variables (measured questionnaire items) that serve as indicators of the latent variables. Circles represent error terms or residuals associated with the observed variables. Single-headed arrows indicate hypothesized directional relationships (pathways) between latent variables and from latent variables to observed variables, with standardized path coefficients labeled on the arrows.

## Discussion

This study revealed that nurses' knowledge regarding bowel preparation for colonoscopy was suboptimal, whereas attitudes were generally positive and practices were at a moderate level. Prior training on bowel preparation emerged as a common independent factor significantly associated with all three KAP dimensions. SEM further showed associations consistent with the hypothesized KAP framework, with knowledge directly associated with practice and an additional indirect association between knowledge and practice through attitude. These findings identify knowledge gaps as a potential target for future educational interventions, whose effects on bowel preparation-related practice should be evaluated in prospective or interventional studies.

No directly comparable study focusing specifically on nurses' KAP regarding bowel preparation for colonoscopy was identified. However, previous KAP studies on colorectal cancer screening among healthcare workers provide useful context. Compared with previous KAP studies conducted among healthcare professionals in colorectal cancer or colonoscopy care, the knowledge level observed in our sample was relatively low. A Chinese multicenter study reported moderate knowledge, positive attitudes, and relatively good practice toward early gastrointestinal cancer among healthcare workers (24), while studies among primary care providers showed variable knowledge and generally favorable attitudes toward colorectal cancer screening (25, 26). The lower knowledge level in our sample may be partly explained by the high proportion of participants from non-endoscopy departments, limited access to procedure-specific training, and the more technical nature of bowel preparation assessment compared with general colorectal cancer screening knowledge. Nevertheless, the generally positive attitude observed in this study was consistent with previous findings among healthcare professionals (25–27). These results suggest that future interventions should prioritize knowledge deficits and practice gaps, particularly in operational aspects of bowel preparation management.

At the knowledge level, the main deficits were observed in the BBPS adequacy criterion, bowel-cleansing agents, dietary restrictions, and risk factors for inadequate bowel preparation. These gaps suggest that nurses' knowledge deficiency was not limited to general awareness but was concentrated in practical and quality-control knowledge. Calderwood and Jacobson demonstrated that BBPS scores of ≥6 reliably predict adequate bowel cleansing (28). Current guidelines emphasize enhanced instructions, split-dose regimens, low-fiber or low-residue diets, and standardized assessment of preparation quality (4). These persistent knowledge gaps underscore the need to incorporate standardized quality assessment tools and evidence-based regimens into routine nursing education.

Attitudes were generally positive, but nurses' perceived role in bowel preparation was less favorable than other attitude items. This finding suggests that some nurses may still regard bowel preparation primarily as physician-directed instruction or patient self-management, rather than as an important component of nursing-led quality improvement. Similar role-related barriers have been reported in other preventive and supportive oncology care contexts. For example, a narrative review of nurses' roles in colorectal cancer prevention emphasized that nurses may contribute to risk assessment, education, screening promotion, and follow-up support, but that these roles are often inconsistently implemented across clinical settings (29). In addition, studies of oncology nurses have shown that positive attitudes do not necessarily translate into practice when nurses lack clear guidelines, multidisciplinary support, or formal role expectations (30, 31). Therefore, insufficient role recognition may weaken nurses' active engagement in bowel preparation management even when they acknowledge its clinical importance.

In the practice domain, the weakest areas centered on individualized guidance for high-risk patients, documentation of bowel cleanliness status, and proactive reporting of inadequate preparation to physicians. These findings indicate a substantial gap between providing routine information to patients and implementing a closed-loop management system that includes assessment, documentation, feedback, and follow-up. In clinical practice, bowel preparation should extend beyond laxative instruction to include risk assessment, tailored education, preparation-quality evaluation, documentation, and timely feedback for inadequate preparation. Because inadequate bowel preparation increases missed lesions and repeat colonoscopy, whereas reinforced education improves preparation adequacy and related outcomes (8, 32, 33), deficiencies in documentation and reporting may disrupt continuity between nursing education, endoscopic assessment, and subsequent management. Comparable implementation gaps have been observed in other nursing contexts where knowledge and positive attitudes did not automatically translate into comprehensive practice without appropriate workflow integration and system support (30).

Multivariable regression further emphasized the consistent association between prior training and all three KAP dimensions. Nurses who had received bowel preparation-related training in the past year had significantly higher knowledge, attitude, and practice scores. This finding is consistent with prior evidence that structured education improves healthcare workers' KAP and that reinforced patient education improves bowel preparation quality (8–10, 24). Several mechanisms may potentially explain the observed association between prior training and KAP scores, including greater exposure to evidence-based knowledge, stronger professional role recognition, and more standardized clinical practices. This is particularly relevant in the Chinese clinical context, where national guidance has emphasized standardized bowel preparation protocols and patient instruction (18), yet training related to bowel preparation is not uniformly embedded across nursing departments. Departmental differences also deserve attention. Nurses in the endoscopy center showed higher knowledge scores than those in general surgery or other departments, which may be related to greater exposure to colonoscopy workflows and more frequent access to specialized guidance. The finding that nurses in tertiary hospitals had lower knowledge and practice scores should be interpreted cautiously. It may reflect sample composition, heavier workload, more fragmented role division, or self-report bias rather than genuinely poorer institutional quality.

The correlation and SEM results provide further support for the KAP framework. Knowledge was positively associated with both attitude and practice, and attitude was also positively associated with practice, consistent with the pattern proposed by the KAP theoretical framework (13, 34). Notably, the direct association between knowledge and practice was stronger than the indirect association through attitude, suggesting that practical competence and procedural knowledge may be particularly important for bowel preparation management. This finding is compatible with the technical nature of bowel preparation, where procedural knowledge may be particularly relevant to appropriate bowel preparation-related practice. However, the significant indirect association through attitude indicates that role recognition and professional motivation may be relevant to the observed relationship between knowledge and practice. Given the cross-sectional design, these structural associations should not be interpreted as evidence of temporal or causal relationships.

Several findings of this study may have clinical and managerial implications. First, considering the prominent association between training and KAP scores, hospitals may consider developing standardized nurse-oriented bowel preparation training programs and extending them across relevant departments, especially non-endoscopy units. Second, such training could prioritize the gaps identified in this study, including BBPS assessment, risk stratification, individualized guidance, diet and laxative regimens, and patient adherence management. Third, bowel preparation education, documentation, reporting, and feedback may need to be better integrated into routine nursing workflows, with appropriate adaptation to local staffing levels and hospital resources. Finally, nurses could be more actively involved in colonoscopy quality-improvement teams, rather than being positioned solely as executors of instructions. However, the effectiveness of these strategies should be further evaluated in longitudinal studies or intervention-based research.

This study has limitations. First, the cross-sectional design precludes causal inference. Second, convenience sampling and online questionnaire distribution may have introduced selection bias. Third, all KAP dimensions were assessed using self-reported data collected at a single time point, which may have introduced recall bias, social desirability bias, and common method variance. Although Harman's single-factor analysis did not indicate dominance by a single common factor, residual common method bias cannot be completely excluded. Fourth, although participants were recruited from eight hospitals, no hospital-specific identifier was retained in the analytic dataset because a unified anonymous electronic questionnaire was used. Therefore, potential within-hospital clustering could not be formally assessed or adjusted for, and future multicenter studies should retain anonymized site identifiers to enable cluster-aware analyses. Fifth, the sample was restricted to one province or region, which may limit generalizability. Sixth, although the questionnaire underwent pilot testing and reliability evaluation, it was developed by the research team and requires further validation in larger and more diverse samples. Seventh, although the SEM supported the hypothesized KAP pathway, the model was based on cross-sectional data and cannot establish a true causal sequence. In addition, some knowledge items combined awareness of a clinical tool with knowledge of a specific criterion (e.g., the BBPS adequacy threshold), which may have increased item complexity. Future studies should further refine, separate, and standardize such items and evaluate their psychometric performance.

In conclusion, this study identified insufficient knowledge, generally positive attitudes, and moderate practice regarding bowel preparation for colonoscopy, particularly for gaps in quality assessment, individualized guidance, and closed-loop management. The observed associations were consistent with the KAP theoretical framework and suggest that standardized training and workflow-level support warrant further evaluation as potential strategies for addressing the identified knowledge and practice gaps.

## Data Availability

The original contributions presented in the study are included in the article/Supplementary material, further inquiries can be directed to the corresponding author.
