# Supplementary material for: Knowledge, attitudes, and practices of nurses regarding bowel preparation for colonoscopy: a structural equation modeling analysis
Source: Front Public Health. 2026 Sep 14;14:1921528. doi: 10.3389/fpubh.2026.1921528 (PMC13616805; doi:10.3389/fpubh.2026.1921528)
Supplement: Supplementary file 1 [file Table_1.docx]

**Table S1. Factors associated with knowledge scores**

| **Variable** | **Univariable linear regression analysis** |  | **Multivariable linear regression analysis** |  |
| --- | --- | --- | --- | --- |
|  | **β (95%CI)** | **P** | **β (95%CI)** | **P** |
| **Sex** |  |  |  |  |
| Male | Reference | — | — | — |
| Female | -0.759 (-2.068 to 0.550) | 0.255 | — | — |
| **Age, years** |  |  |  |  |
| ≤34 | Reference | — | — | — |
| 35–39 | -0.176 (-1.338 to 0.987) | 0.767 | — | — |
| ≥40 | 0.573 (-0.704 to 1.850) | 0.378 | — | — |
| **Educational level** |  |  |  |  |
| Junior college | Reference | — | Reference | — |
| Bachelor’s degree | -1.150 (-3.069 to 0.770) | 0.240 | -0.840 (-2.585 to 0.905) | 0.345 |
| Master’s degree or above | 0.859 (-1.565 to 3.283) | 0.486 | 0.630 (-1.602 to 2.861) | 0.579 |
| **Department** |  |  |  |  |
| Endoscopy center | Reference | — | Reference | — |
| Department of Gastroenterology | -1.960 (-3.666 to -0.250) | 0.025 | -1.505 (-3.051 to 0.041) | 0.057 |
| General Surgery Department | -4.630 (-6.673 to -2.589) | <0.001 | -2.812 (-4.681 to -0.943) | 0.003 |
| Other departments | -2.810 (-4.484 to -1.143) | 0.001 | -2.071 (-3.611 to -0.532) | 0.008 |
| **Work experience, years** |  |  |  |  |
| ≤5 | Reference | — | Reference | — |
| 5–10 | -0.217 (-1.792 to 1.358) | 0.787 | -0.548 (-1.963 to 0.867) | 0.447 |
| 11–20 | -1.220 (-2.608 to 0.161) | 0.083 | -1.480 (-2.772 to -0.188) | 0.025 |
| ≥21 | 1.040 (-0.759 to 2.837) | 0.256 | 0.911 (-0.724 to 2.545) | 0.274 |
| **Professional title** |  |  |  |  |
| Junior title or below | Reference | — | — | — |
| Intermediate title | -0.264 (-1.474 to 0.945) | 0.668 | — | — |
| Senior title, including associate senior title | 0.331 (-1.254 to 1.917) | 0.682 | — | — |
| **Hospital type** |  |  |  |  |
| Public hospital | Reference | — | — | — |
| Private hospital | 1.250 (-0.852 to 3.358) | 0.243 | — | — |
| **Hospital level** |  |  |  |  |
| Non-tertiary hospital | Reference | — | Reference | — |
| Tertiary hospital | -2.020 (-3.521 to -0.522) | 0.008 | -1.795 (-3.125 to -0.465) | 0.008 |
| **Teaching responsibilities** |  |  |  |  |
| Yes | Reference | — | — | — |
| No | -0.308 (-1.313 to 0.697) | 0.547 | — | — |
| **Research responsibilities** |  |  |  |  |
| Yes | Reference | — | Reference | — |
| No | -1.350 (-2.444 to -0.257) | 0.016 | -1.102 (-2.129 to -0.075) | 0.035 |
| **Monthly colonoscopy-related nursing frequency in the past year, cases** |  |  |  |  |
| <10 | Reference | — | — | — |
| 10–20 | 0.222 (-1.337 to 1.782) | 0.779 | — | — |
| 21–30 | 0.903 (-0.617 to 2.423) | 0.243 | — | — |
| >30 | 0.443 (-1.282 to 2.168) | 0.614 | — | — |
| **Training related to bowel preparation during the past year** |  |  |  |  |
| Yes | Reference | — | Reference | — |
| No | -4.480 (-5.393 to -3.569) | <0.001 | -4.097 (-5.006 to -3.188) | <0.001 |

Note: β, regression coefficient; CI, confidence interval. Reference indicates the reference category in the regression model. Variables not included in the multivariable model are indicated by “—”.

**Table S2. Factors associated with Attitude scores**

| **Variable** | **Univariable linear regression analysis** |  | **Multivariable linear regression analysis** |  |
| --- | --- | --- | --- | --- |
|  | **β (95%CI)** | **P** | **β (95%CI)** | **P** |
| **Sex** |  |  |  |  |
| Male | Reference | — | — | — |
| Female | -0.999 (-2.220 to 0.222) | 0.108 | — | — |
| **Age, years** |  |  |  |  |
| ≤34 | Reference | — | — | — |
| 35–39 | -0.441 (-1.527 to 0.644) | 0.425 | — | — |
| ≥40 | 0.248 (-0.944 to 1.441) | 0.683 | — | — |
| **Educational level** |  |  |  |  |
| Junior college | Reference | — | Reference | — |
| Bachelor’s degree | -0.524 (-2.319 to 1.271) | 0.566 | -0.238 (-1.847 to 1.372) | 0.772 |
| Master’s degree or above | 1.300 (-0.970 to 3.564) | 0.261 | 0.734 (-1.283 to 2.751) | 0.475 |
| **Department** |  |  |  |  |
| Endoscopy center | Reference | — | Reference | — |
| Department of Gastroenterology | 0.139 (-1.487 to 1.765) | 0.867 | 1.024 (-0.442 to 2.491) | 0.17 |
| General Surgery Department | -1.360 (-3.308 to 0.579) | 0.168 | 0.785 (-1.020 to 2.589) | 0.393 |
| Other departments | -1.100 (-2.690 to 0.489) | 0.174 | 0.169 (-1.298 to 1.635) | 0.821 |
| **Work experience, years** |  |  |  |  |
| ≤5 | Reference | — | — | — |
| 5–10 | -0.203 (-1.685 to 1.279) | 0.788 | — | — |
| 11–20 | -0.589 (-1.892 to 0.714) | 0.374 | — | — |
| ≥21 | 0.863 (-0.829 to 2.554) | 0.317 | — | — |
| **Professional title** |  |  |  |  |
| Junior title or below | Reference | — | — | — |
| Intermediate title | -0.163 (-1.291 to 0.964) | 0.776 | — | — |
| Senior title, including associate senior title | 0.805 (-0.673 to 2.283) | 0.285 | — | — |
| **Hospital type** |  |  |  |  |
| Public hospital | Reference | — | — | — |
| Private hospital | 1.520 (-0.443 to 3.484) | 0.129 | — | — |
| **Hospital level** |  |  |  |  |
| Non-tertiary hospital | Reference | — | — | — |
| Tertiary hospital | -1.050 (-2.462 to 0.357) | 0.143 | — | — |
| **Teaching responsibilities** |  |  |  |  |
| Yes | Reference | — | — | — |
| No | -0.489 (-1.427 to 0.448) | 0.305 | — | — |
| **Research responsibilities** |  |  |  |  |
| Yes | Reference | — | — | — |
| No | -0.481 (-1.509 to 0.547) | 0.358 | — | — |
| **Monthly colonoscopy-related nursing frequency in the past year, cases** |  |  |  |  |
| <10 | Reference | — | — | — |
| 10–20 | -0.389 (-1.847 to 1.069) | 0.600 | — | — |
| 21–30 | -0.349 (-1.770 to 1.072) | 0.629 | — | — |
| >30 | 0.233 (-1.380 to 1.846) | 0.776 | — | — |
| **Training related to bowel preparation during the past year** |  |  |  |  |
| Yes | Reference | — | Reference | — |
| No | -2.770 (-3.683 to -1.866) | <0.001 | -1.052 (-2.000 to -0.104) | 0.030 |
| **Knowledge score** | 0.432 (0.349 to 0.516) | <0.001 | 0.381 (0.287 to 0.476) | <0.001 |

Note: β, regression coefficient; CI, confidence interval. Reference indicates the reference category in the regression model. Variables not included in the multivariable model are indicated by “—”.

**Table S3. Factors associated with Practice scores**

| **Variable** | **Univariable linear regression analysis** |  | **Multivariable linear regression analysis** |  |
| --- | --- | --- | --- | --- |
|  | **β (95%CI)** | **P** | **β (95%CI)** | **P** |
| **Knowledge score** | 0.991 (0.897 to 1.086) | <0.001 | 0.781 (0.668 to 0.894) | <0.001 |
| **Attitude score** | 0.690 (0.560 to 0.820) | <0.001 | 0.223 (0.114 to 0.331) | <0.001 |
| **Sex** |  |  |  |  |
| Male | Reference | — | — | — |
| Female | -0.830 (-2.618 to 0.957) | 0.362 | — | — |
| **Age, years** |  |  |  |  |
| ≤34 | Reference | — | Reference | — |
| 35–39 | -1.680 (-3.253 to -0.103) | 0.037 | -1.483 (-3.006 to 0.041) | 0.056 |
| ≥40 | 0.459 (-1.271 to 2.189) | 0.602 | 0.515 (-1.377 to 2.406) | 0.593 |
| **Educational level** |  |  |  |  |
| Junior college | Reference | — | — | — |
| Bachelor’s degree | -1.110 (-3.747 to 1.518) | 0.406 | — | — |
| Master’s degree or above | 0.528 (-2.797 to 3.854) | 0.755 | — | — |
| **Department** |  |  |  |  |
| Endoscopy center | Reference | — | Reference | — |
| Department of Gastroenterology | -1.960 (-4.308 to 0.388) | 0.102 | -0.039 (-1.664 to 1.586) | 0.962 |
| General Surgery Department | -5.420 (-8.228 to -2.615) | <0.001 | -0.071 (-2.022 to 1.880) | 0.943 |
| Other departments | -2.990 (-5.282 to -0.690) | 0.011 | 0.272 (-1.327 to 1.871) | 0.738 |
| **Work experience, years** |  |  |  |  |
| ≤5 | Reference | — | Reference | — |
| 5–10 | -2.200 (-4.350 to -0.044) | 0.045 | -1.787 (-3.275 to -0.299) | 0.019 |
| 11–20 | -2.440 (-4.331 to -0.545) | 0.012 | -0.767 (-2.626 to 1.092) | 0.418 |
| ≥21 | -0.544 (-3.002 to 1.914) | 0.664 | -2.338 (-4.789 to 0.113) | 0.061 |
| **Professional title** |  |  |  |  |
| Junior title or below | Reference | — | — | — |
| Intermediate title | -0.622 (-2.273 to 1.028) | 0.459 | — | — |
| Senior title, including associate senior title | -0.876 (-3.040 to 1.287) | 0.426 | — | — |
| **Hospital type** |  |  |  |  |
| Public hospital | Reference | — | — | — |
| Private hospital | 0.623 (-2.254 to 3.500) | 0.671 | — | — |
| **Hospital level** |  |  |  |  |
| Non-tertiary hospital | Reference | — | Reference | — |
| Tertiary hospital | -3.480 (-5.511 to -1.441) | <0.001 | -1.411 (-2.803 to -0.020) | 0.047 |
| **Teaching responsibilities** |  |  |  |  |
| Yes | Reference | — | — | — |
| No | 0.145 (-1.227 to 1.517) | 0.835 | — | — |
| **Research responsibilities** |  |  |  |  |
| Yes | Reference | — | Reference | — |
| No | -1.570 (-3.066 to -0.076) | 0.040 | -0.515 (-1.534 to 0.504) | 0.321 |
| **Monthly colonoscopy-related nursing frequency in the past year, cases** |  |  |  |  |
| <10 | Reference | — | — | — |
| 10–20 | -0.801 (-2.926 to 1.324) | 0.459 | — | — |
| 21–30 | 0.689 (-1.382 to 2.760) | 0.513 | — | — |
| >30 | 0.026 (-2.324 to 2.377) | 0.983 | — | — |
| **Training related to bowel preparation during the past year** |  |  |  |  |
| Yes | Reference | — | Reference | — |
| No | -6.290 (-7.530 to -5.060) | <0.001 | -2.297 (-3.335 to -1.260) | <0.001 |

Note: β, regression coefficient; CI, confidence interval. Reference indicates the reference category in the regression model. Variables not included in the multivariable model are indicated by “—”.

**Table S4. Correlation analysis**

| **Spearman** | **Knowledge** | **Attitude** | **Practice** |
| --- | --- | --- | --- |
| **Knowledge** | 1.000 |  |  |
| **Attitude** | 0.434 (P<0.001) | 1.000 |  |
| **Practice** | 0.707 (P<0.001) | 0.471 (P<0.001) | 1.000 |

**Table S5. SEM fit indicators**

| **Indicators** | **Reference** | **Results** |
| --- | --- | --- |
| RMSEA | <0.080 | 0.071 |
| CMIN/DF | <5 | 2.941 |
| TLI | >0.900 | 0.911 |
| CFI | >0.900 | 0.92 |

**Note:** SEM, structural equation modeling; RMSEA, root mean square error of approximation; CMIN/DF, chi-square/degrees of freedom ratio; TLI, Tucker–Lewis index; CFI, comparative fit index.

**Table S6. Unstandardized and standardized path coefficients of the structural equation model**

| **Path category** | **Path** | **Estimate** | **SE** | **CR** | **P** | **Standardized β** | **95% CI forβ** |
| --- | --- | --- | --- | --- | --- | --- | --- |
| Structural path | Knowledge → Attitude | 0.576 | 0.069 | 8.376 | <0.001 | 0.491 | 0.407–0.584 |
| Structural path | Knowledge → Practice | 1.222 | 0.116 | 10.535 | <0.001 | 0.707 | 0.626–0.780 |
| Structural path | Attitude → Practice | 0.205 | 0.066 | 3.093 | 0.002 | 0.139 | 0.045–0.230 |
| Measurement path | Knowledge → K1 | 1 | — | — | — | 0.704 | 0.628–0.764 |
| Measurement path | Knowledge → K2 | 1.161 | 0.077 | 15.11 | <0.001 | 0.804 | 0.747–0.847 |
| Measurement path | Knowledge → K3 | 1.154 | 0.088 | 13.09 | <0.001 | 0.697 | 0.636–0.752 |
| Measurement path | Knowledge → K4 | 1.017 | 0.076 | 13.306 | <0.001 | 0.706 | 0.655–0.758 |
| Measurement path | Knowledge → K5 | 1.288 | 0.087 | 14.805 | <0.001 | 0.791 | 0.740–0.830 |
| Measurement path | Knowledge → K6 | 1.177 | 0.077 | 15.246 | <0.001 | 0.821 | 0.777–0.858 |
| Measurement path | Knowledge → K7 | 1.224 | 0.08 | 15.392 | <0.001 | 0.83 | 0.779–0.869 |
| Measurement path | Knowledge → K8 | 1.252 | 0.083 | 15.057 | <0.001 | 0.81 | 0.759–0.854 |
| Measurement path | Knowledge → K9 | 1.244 | 0.084 | 14.766 | <0.001 | 0.796 | 0.736–0.844 |
| Measurement path | Knowledge → K10 | 1.159 | 0.082 | 14.204 | <0.001 | 0.764 | 0.698–0.813 |
| Measurement path | Attitude → A1 | 1.02 | 0.073 | 13.952 | <0.001 | 0.685 | 0.607–0.756 |
| Measurement path | Attitude → A2 | 0.84 | 0.094 | 8.95 | <0.001 | 0.459 | 0.349–0.574 |
| Measurement path | Attitude → A3 | 0.987 | 0.06 | 16.497 | <0.001 | 0.79 | 0.651–0.856 |
| Measurement path | Attitude → A4 | 1.002 | 0.058 | 17.372 | <0.001 | 0.83 | 0.767–0.878 |
| Measurement path | Attitude → A5 | 1.101 | 0.059 | 18.797 | <0.001 | 0.881 | 0.826–0.922 |
| Measurement path | Attitude → A6 | 0.998 | 0.064 | 15.569 | <0.001 | 0.751 | 0.626–0.830 |
| Measurement path | Attitude → A7 | 1.125 | 0.064 | 17.485 | <0.001 | 0.826 | 0.776–0.873 |
| Measurement path | Attitude → A8 | 1.125 | 0.07 | 16.082 | <0.001 | 0.772 | 0.698–0.840 |
| Measurement path | Attitude → A9 | 1 | — | — | — | 0.77 | 0.638–0.843 |
| Measurement path | Practice → P1 | 1 | — | — | — | 0.73 | 0.665–0.782 |
| Measurement path | Practice → P2 | 1.318 | 0.094 | 14.066 | <0.001 | 0.727 | 0.672–0.780 |
| Measurement path | Practice → P3 | 0.644 | 0.08 | 8.075 | <0.001 | 0.427 | 0.318–0.517 |
| Measurement path | Practice → P4 | 0.893 | 0.069 | 13.016 | <0.001 | 0.68 | 0.596–0.742 |
| Measurement path | Practice → P5 | 1.079 | 0.072 | 14.982 | <0.001 | 0.785 | 0.717–0.834 |
| Measurement path | Practice → P6 | 1.343 | 0.079 | 17.044 | <0.001 | 0.873 | 0.839–0.899 |
| Measurement path | Practice → P7 | 1.421 | 0.087 | 16.247 | <0.001 | 0.841 | 0.786–0.878 |
| Measurement path | Practice → P8 | 1.365 | 0.082 | 16.577 | <0.001 | 0.852 | 0.805–0.890 |

Note: Estimate represents the unstandardized path coefficient or factor loading. Standardized β represents the standardized coefficient. BC CI, bias-corrected bootstrap confidence interval. Bootstrap confidence intervals were calculated using 1,000 resamples. Reference indicators were fixed to 1.000 for model identification; therefore, SE, CR, and P values were not estimated for these paths.
